# Use of Hashtags related to Covid-19 infodemics by bot accounts

**DOI:** 10.1093/eurpub/ckac131.172

**Published:** 2022-10-25

**Authors:** V Suarez-Lledo, B Ramos-Fiol, ME Ortega-Martin, J Carretero-Bravo, J Alvarez-Galvez

**Affiliations:** 1 Department of Biomedicine, Biotechnology and Public Health, University of Cadiz, Cádiz, Spain; 2 Clinical Medicine and Health Public, University of Granada, Granada, Spain

## Abstract

**Background:**

Along with the Covid-19 pandemic we need to fight an ‘infodemic'. Some of the most widespread social media platforms such as Facebook, Instagram and Twitter have implemented policies to combat the spread of misinformation about Covid. However, the online ecosystem is still full of health myths, hoaxes, and fake news that-either consciously or unconsciously-is propagated by social media users with different purposes, messages that can lead to attitudinal and behavioral changes which might result in inadequate health decision making

**Methods:**

We use Twitter Stream API to collect tweets about Covid-19 during the early outbreak. Then we filtered those tweets with hashtags related to three infodemic topics: 5g, bill gates, UV and hydroxychloroquine. Then, we use Botometer to obtain the probability that each account is a bot or not. We use bot classification along with network analysis (Louvain community detection) to delve into the subtopics and the use of hashtags.

**Results:**

The resulting data collection contains ∼14M tweets from ∼285K of different Twitter accounts. We selected only tweets written in English. Regarding 5G, the most important communities link China with the virus, are about “democratshateamerica” or conspiracy theories. Tweets about Bill Gates contain hashtags about Trump, America, or mention the batflu. Communities related with UV are about Trump disinfectant, or pointing out that tv channels spread fake news. Those tweets that mention hydroxychloroquine mostly contain hashtags that mention qanon or maga content.

**Conclusions:**

In this paper, we analyze the use of hashtags by accounts classified as bots. Using Louvain community detection we identify co-occurring hashtags. Using social network analysis we identify which hashtags are the most important within the conversation.

**Key messages:**

• We identify several communities around most important infodemic topics. Bots activity in most of the cases is about political content than spreading health misinformation.

• This method allows to find subtopics based on the use of hashtags. Which allow public health policies to prevent the spread of infodemics.

